# Dexrazoxane may prevent doxorubicin-induced DNA damage via depleting both Topoisomerase II isoforms

**DOI:** 10.1186/1471-2407-14-842

**Published:** 2014-11-18

**Authors:** Shiwei Deng, Tiandong Yan, Cathleen Jendrny, Andrea Nemecek, Mladen Vincetic, Ute Gödtel-Armbrust, Leszek Wojnowski

**Affiliations:** Institute of Pharmacology, Medical Center of the University Mainz, Obere Zahlbacher Str. 67, D-55131 Mainz, Germany

## Abstract

**Background:**

The bisdioxopiperazine dexrazoxane (DRZ) prevents anthracycline-induced heart failure, but its clinical use is limited by uncertain cardioprotective mechanism and by concerns of interference with cancer response to anthracyclines and of long-term safety.

**Methods:**

We investigated the effects of DRZ on the stability of topoisomerases IIα (TOP2A) and IIβ (TOP2B) and on the DNA damage generated by poisoning these enzymes by the anthracycline doxorubicin (DOX).

**Results:**

DRZ given i.p. transiently depleted in mice the predominant cardiac isoform Top2b. The depletion was also seen in H9C2 cardiomyocytes and it was attenuated by mutating the bisdioxopiperazine binding site of TOP2B. Consistently, the accumulation of DOX-induced DNA double strand breaks (DSB) by wild-type, although not by mutant TOP2B, was reduced by DRZ. In contrast, the DRZ analogue ICRF-161, which is capable of iron chelation but not of TOP2B binding and cardiac protection, did not deplete TOP2B and did not prevent the accumulation of DOX-induced DSB. TOP2A, re-expressed in cultured cardiomyocytes by fresh serum, was depleted by DRZ along with TOP2B. DRZ depleted TOP2A also from fibrosarcoma-derived cells, but not from lung cancer-derived and human embryo-derived cells. DRZ-mediated TOP2A depletion reduced the accumulation of DOX-induced DSB.

**Conclusions:**

Taken together, our data support a model of anthracycline-induced heart failure caused by TOP2B-mediated DSB and of its prevention by DRZ via TOP2B degradation rather than via iron chelation. The depletion of TOP2B and TOP2A suggests an explanation for the reported DRZ interference with cancer response to anthracyclines and for DRZ side-effects.

## Background

Heart failure (HF) is a foremost side-effect of oncological therapies, most prominently those comprising anthracyclines, which are administered to an estimated 50% of cancer patients. Certain reductions in heart failure rates have been achieved by capping the maximal doses of anthracyclines and by changing their administration schedules. Nevertheless, heart failure continues to affect up to 20% of anthracycline-treated patients [1]. The retrospectively identified individual risk factors such as young or old age, female sex, cardiovascular co-morbidities, certain co-therapies, and gene variants are currently of limited help. This is due to the lack of prospective clinical validation and to the scarcity of equipotent anthracycline-free treatment alternatives. Particularly problematic is the high HF risk in anthracycline-treated children and in the elderly attributed, respectively, to the long life expectancy and cardiac co-morbidities.

The bisdioxopiperazine dexrazoxane (DRZ, ICRF-187) reduces the incidence of anthracycline-induced heart failure by 80% and is the only drug approved for its prevention [2]. Despite its impressive clinical effect, DRZ is indicated only in patients with metastatic (in the EU also with advanced) breast cancer who have already received 300 mg/m^2^ doxorubicin (in the EU also epirubicin, 540 mg/m^2^). Anecdotal evidence indicates a limited off-label use of DRZ in cancer patients with cardiac co-morbidities. DRZ is currently not approved for use in children and adolescents. Besides complex patent ownership and marketing history, the limited indication and use of DRZ reflect the persisting concerns of interference with antitumor efficacy of anthracyclines [3], of induction of secondary malignancies, and of myelodysplastic syndrome [4], although recent meta-analyses [5] do not support these concerns. One study even reported prolonged, almost doubled, survival of doxorubicin (DOX) responders co-treated with DRZ [6].

The clinical acceptance of DRZ has also suffered from the incomplete understanding of its pharmacology. The cardioprotective effect of DRZ is usually attributed to iron chelating by ADR-925, an EDTA-like product of the enzymatic DRZ hydrolysis. This is consistent with the hypothesis of heart failure arising from iron-dependent oxidative stress triggered by “redox cycling” of anthracyclines. However, ADR-925 applied directly does not protect cardiomyocytes against anthracyclines [7]. Conversely, the inhibition of the enzymatic DRZ conversion to ADR-925 does not abolish the cardioprotective effect [8]. Furthermore, cardioprotective activities of DRZ analogues are unrelated to the rates of formation of their iron-chelating (i.e. ADR-925-like) metabolites [9]. Finally, the oxidative stress theory of anthracycline cardiotoxicity has been questioned, since oxidative stress is detectable in the heart following supra-clinical, but not clinically-relevant anthracycline doses [10]. Accordingly, neither antioxidants [11] nor iron chelators other than DRZ [12, 13] prevent anthracycline-induced HF in cancer patients.

On the other hand, several investigators have observed depletion of the anthracycline target topoisomerase IIβ (TOP2B) in cells treated with DRZ or its analogues [14–16]. Lyu et al. [15] reported that DRZ-induced Top2b depletion prevented anthracycline-induced DNA damage in cardiomyocyte-derived H9C2 cells. Based on these observations, Lyu et al. proposed that DRZ prevents anthracycline-induced cardiomyocyte damage via TOP2B depletion. Importantly, this model also implicates that the anthracycline-induced cardiotoxicity is primarily driven by TOP2B-mediated DNA damage. In support of these findings, a recent study in cardiomyocyte-specific knockouts suggested the involvement of Top2b in DOX-induced cardiotoxicity [17]. Importantly, Top2b deletion prevented DOX-driven mitochondrial damage, implicating this enzyme as a common denominator of the mitochondrial and DNA damage-based models of anthracycline cardiotoxicity.

The TOP2B-centered model of anthracycline-induced heart failure and its prevention by DRZ clearly needs further verification when weighed against the body of literature supporting the original oxidative stress hypothesis. Furthermore, this model is incompatible with some other findings. Thus, it is inexplicable why DRZ fully abolished DOX-induced DNA double strand breaks (DSB) from cultured cardiomyocytes, as DRZ left unchanged the expression level of the sister isoform Topoisomerase IIα (TOP2A) [15], an established DOX target. The TOP2A expression itself raises concerns regarding the validity of the cells used as a cardiomyocyte model, as it is usually expressed in proliferating and not post-mitotic cells [18]. On the other hand, DRZ has been recently reported to deplete TOP2A from tumor cells [14]. This depletion was proteasome-independent but it was associated with a strong reduction of TOP2A mRNA, whereas TOP2B mRNA remained unchanged. These results suggested that DRZ may deplete both TOP2B and TOP2A via different, transcriptional and proteolytic mechanisms, respectively. The verification of this possibility is important not only in organ protection but also for tumor treatments. Indeed, tumor TOP2A expression is a strong predictor of the therapeutic response to anthracyclines [19] and its reduction by DRZ could be deleterious. Therefore, we set out to characterize the response of TOP2 isozymes to DRZ in cardiac and tumor cells.

## Methods

### Drugs and antibodies

DRZ was purchased from Chiron (Amsterdam, The Netherlands), DOX was from Pfizer (Karlsruhe, Germany). ICRF-161 and ICRF-193 were kindly provided by Dr. Annemette V. Thougaard (TopoTarget A/S, Copenhagen, Denmark). The following antibodies were used in Western blot: TOP2A/B (1: 200) and TOP2A (1:2,000) from StressGen, TOP2B (1:2,000) from BD Pharmingen, γ-H2AX (1:500) from Abcam, GFP (1:5,000) from Clontech, GAPDH (1:10,000) from Santa Cruz Biotechnology, α-tubulin (1:5,000) from Dianova (Germany) and β-tubulin (1:100,000) from Sigma.

### Cell culture

HTETOP cells express TOP2A exclusively from a transgene under the control of a tetracycline-repressive promoter, have been previously established [20]. HTETOP and the parental HT1080 cells were provided by Dr. Andrew C.G. Porter (Centre for Haematology, Faculty of Medicine, Imperial College London, England) and cultured as previously described [14]. To obtain differentiation, H9C2 cells (provided by Dr. Tomas Simunek, Department of Biochemical Sciences, Charles University in Prague, Czech Republic) were continuously cultured for 14 days: 3 days in 10% FCS medium and the following 11 days in 1% FCS medium. HEK293 cells were obtained from Dr. Christian Mielke (Institute of Clinical Chemistry and Laboratory Diagnostics, Heinrich-Heine-University, Düsseldorf, Germany) and NYH cells were from Dr. Annemette V. Thougaard (TopoTarget A/S, Copenhagen, Denmark).

### Mice and treatment

Wild type C57BL/6 J (B6) mice were purchased from Jackson Lab (Bar Harbor, ME) and they were 8–12 weeks old at the onset of experiment. Mice were injected i.p. with 100 μl of saline (control group, *n* =3) or 200 mg/kg DRZ in the same volume of saline (DRZ group, *n* =9). Animals were sacrificed by cervical dislocation 6, 24 or 48 h after DRZ injection and the whole hearts were isolated and snap-frozen in liquid nitrogen. In parallel experiments, mice were injected with 200 mg/kg DRZ alone (*n* =3), 20 mg/kg DOX (*n* =3) alone or in combination (*n* =3, DRZ was injected 30 min before DOX). Heart tissues were removed 24 h later for Western blot analysis. The mouse experiments described in this paper have been permitted by the Animal Care Committee of Rhineland-Palatinate (Koblenz, Germany) with the permit No. AZ 23 177-07/G 12-1-043.

### Western blot

Heart tissues from control or drug-treated mice were homogenized thoroughly in RIPA buffer containing proteinase inhibitors [14]. The lysate was subsequently incubated for 2 h at 4°C with agitation. Cultured cells were harvested and lysed in RIPA buffer followed by 30 min incubation on ice. Twenty to 50 μg protein samples were separated by SDS-PAGE as described previously [14]. Densitometric analysis of protein bands was performed using the NIH Image J software.

### RNA interfering and transient transfection

siRNA oligos were used to knock down human TOP2B with the target region GCTTAACAATCAAGCCCGT [21]. siRNA oligos against the GFP sequence GGCTACGTCCAGGAGCGCACC were used as non-silencing control. To specifically knock down the endogenous TOP2B without affecting the transfected TOP2B-YFP, three siRNA oligos targeting the 3-UTR of human TOP2B mRNA were applied, since the TOP2B-YFP expression vector encompasses only the open reading frame of TOP2B without 3-UTR. The target sequences for 3-UTR region were as follows: siRNA1: GAAATGTCACGTACTGTCTGA; siRNA2: ACTGTCTGATTGGCTTGTAGA; siRNA3: GCTTGTAGAATTGTTATAGAC. For H9C2 cells, the target sequence of siRNA to knock down rat Top2b was: GAACAAAGCTGGTGTATCA. siRNA oligos were transfected at the final concentration of 50 nM using the jetPRIME^TM^ transfection reagent (Polyplus Transfection SA, Illkirch, France) according to the specifications of the manufacturer.

### Site-directed mutagenesis

The bicistronic vector expressing human TOP2B fused with enhanced yellow fluorescent protein (YFP) at the C-terminal has been described previously [22]. Single amino acid substitution was introduced into this vector with the site-directed mutagenesis Kit (QuikChange, Stratagene). Primers for individual TOP2B mutants are shown in Table 1. The mutated sites are highlighted by italic bold letters. PCR conditions for mutagenesis were according to the manufacturer’s instructions. The introduction of the desired and the absence of off-target mutations was verified by sequencing.

**Table 1:** **Primers for site-directed mutagenesis of human TOP2B**

T65I	forward	cattcttcttcgtcctgata***t***atatattgggtcagtggagc
	reverse	gctccactgacccaatatat***a***tatcaggacgaagaagaatg
Y66F	forward	tcttcgtcctgatacat***t***tattgggtcagtggagc
	reverse	gctccactgacccaata***a***atgtatcaggacgaaga
R178Q	forward	gatgagaaaaaagttacaggtggtc***aa***aatggttatggtgcaaaactttgta
	reverse	tacaaagttttgcaccataaccatt***tt***gaccacctgtaacttttttctcatc
Y181S	forward	aaagttacaggtggtcgtaatggtt***c***tggtgcaaaactttg
	reverse	caaagttttgcacca***g***aaccattacgaccacctgtaacttt
L185F	forward	ggtcgtaatggttatggtgcaaaa***t***tttgtaatattttcagtacaaagt
	reverse	actttgtactgaaaatattacaaa***a***ttttgcaccataaccattacgacc

The mutated sites are highlighted by italic bold letters.

### RNA-Seq data analysis

The raw RNA-Seq data obtained from diverse human or mouse organs were downloaded in Fastq format from Gene Expression Omnibus (GEO) in NCBI (accession number GSE30352) [23]. Original reads were mapped against human (version GRCh37/hg19) or mouse (version NCBI37/mm9) genome with TopHat using gene annotation file as the reference for alignment [24]. The genomic sequence data and gene annotation files were downloaded from UCSC (http://genome.ucsc.edu/). Mapped reads were assembled with the program Cufflinks [24] and the expression levels of individual transcripts were calculated as Fragment Per Kilobase of exon per Million fragments mapped (FPKM).

### Statistical analysis

All experiments were repeated at least three times. Data were expressed as mean ± SE and analyzed with the two-tailed Student’s t-test or one-way ANOVA. *P* <0.05 was considered statistically significant.

## Results

### DRZ depletes cardiac TOP2B protein both *in vitro in*and *vivo*

**Figure 1:**
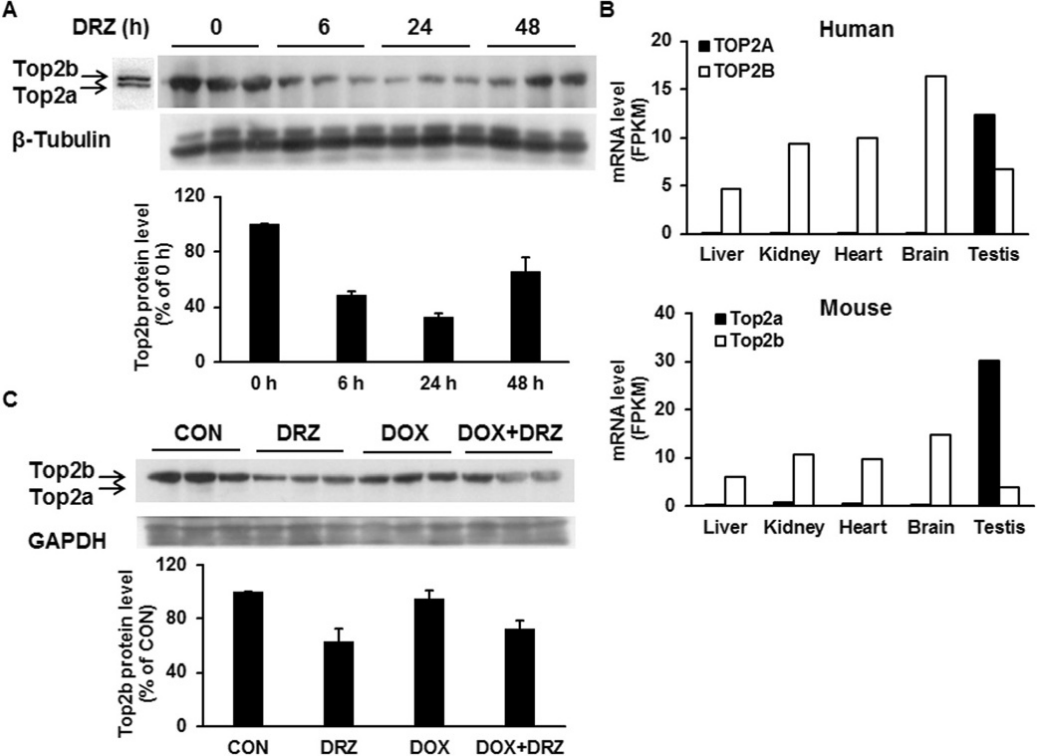
**Cardiac expression of TOP2 isoforms and its response to DRZ. (A)** Top2a/b Western blot of heart tissues from B6 mice injected i.p. with 200 mg/kg DRZ. Whole hearts were removed 6, 24 or 48 h after injection. Western blot was performed with an antibody detecting both Top2a and Top2b under conditions permitting the resolving of these isoforms in H9C2 cells (the leftmost lane). **(B)** TOP2A and TOP2B mRNA expression levels in different human or mouse organs derived from online available RNA-Seq data (NCBI, GEO accession number GSE30352). **(C)** Western blot of heart tissues from B6 mice injected i.p. with 200 mg/kg DRZ and/or 20 mg/kg DOX, DRZ was given 30 min before DOX. Hearts were removed 24 h later for Western blot analysis using an antibody against Top2a and Top2b.

Western blot analysis of mouse heart homogenates revealed a single band with molecular weight corresponding to Top2b (time 0 h, Figure 1A). This was in agreement with TOP2B/Top2b mRNA transcripts accounting for >95% of combined TOP2A and TOP2B transcripts in human or mouse hearts (Figure 1B). To investigate DRZ effects on Top2b protein expression *in vivo*, B6 mice were injected with 200 mg/kg DRZ and heart tissues were removed 6, 24 or 48 h later for Western blot analysis. DRZ treatment clearly depressed Top2b protein expression 6 h after injection, with partial recovery observed at 48 h (Figure 1A). The depletion of Top2b was observed also when DRZ was followed 30 min later by 20 mg/kg DOX given i.p., which modelled the recommended time-course of combining anthracyclines with DRZ in humans (Figure 1C).

**Figure 2:**
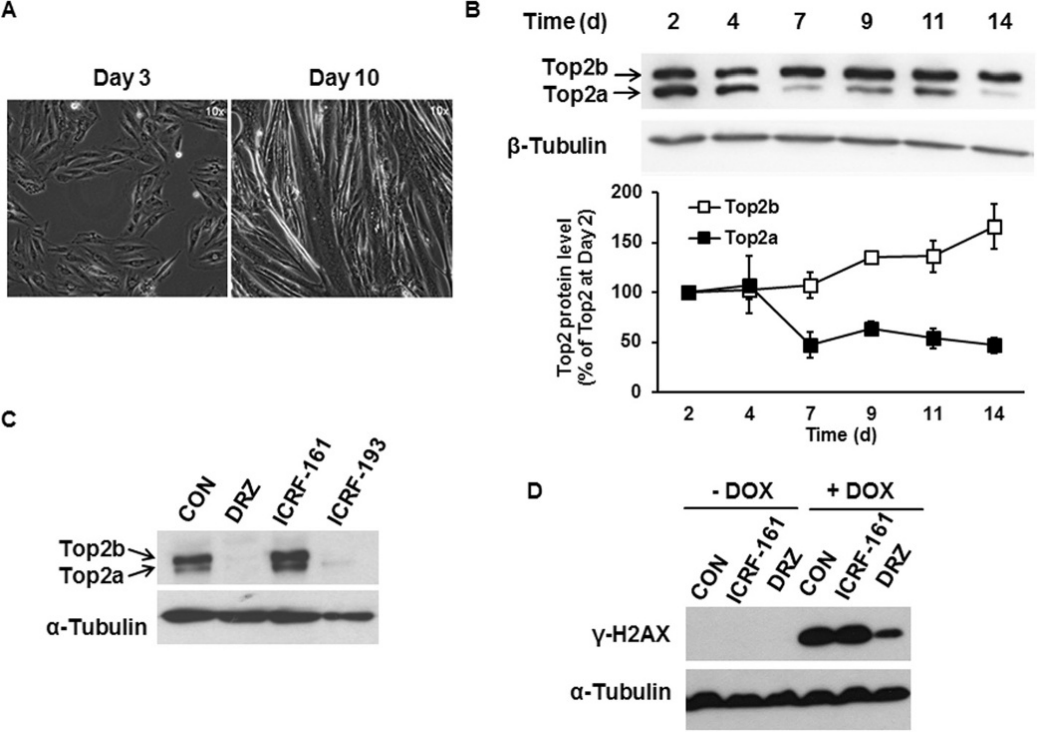
**The expression of Top2 isoforms in differentiated cardiomyocytes and their responses to DRZ. (A)** Differentiation of H9C2 cells into myotubes after 3 and 10 days of culturing assessed by light microscopy. Magnification: 10 fold. **(B)** Western blot analysis of Top2a and Top2b expression in H9C2 cells during the first 14 days of culturing. **(C)** The effect of 24 h incubation with DRZ and its analogues ICRF-161 and ICRF-193 (100 μM each) on Top2 expression in H9C2 cells. B and C are Western blots using antibody reacting with both Top2a and Top2b. In B, band densities of either isoform were divided through those of the time point-matching internal standard β-tubulin and plotted with day 2 Top2/β-tubulin ratio taken as 100%. **(D)** The effect of co-treatment with 100 μM DRZ or with its analogue ICRF-161 on DSB γ-H2AX protein levels induced by 1 μM DOX (24 h).

These *in vivo* data differed from the reported expression of both Top2 isoforms in the cardiomyocyte-derived H9C2, with DRZ selectively depleting Top2b [15]. We therefore assessed Top2 levels as a function of H9C2 differentiation from fibroblast-like cells to multinucleated myotubes, which form only after >10 days of culturing (Figure 2A). Undifferentiated cells expressed both Top2 isoforms (day 2, Figure 2B). The differentiation was accompanied by pronounced decrease of Top2a protein expression and by modest increase of Top2b (Figure 2B). DRZ and the likewise Top2-binding analogue ICRF-193 depleted Top2b from differentiated H9C2 cells, in contrast to the non-binding [25] analogue ICRF-161 (Figure 2C). DRZ, but not ICRF-161 reduced the accumulation of DOX-induced DSB (Figure 2D). These results were suggestive of DRZ capable of reducing DOX-induced DSB in cardiac cells via depletion of the DOX target Top2b, the predominant cardiac Top2 isoform.

### The protective effect of DRZ is mediated by the TOP2B bisdioxopiperazine binding site

**Figure 3:**
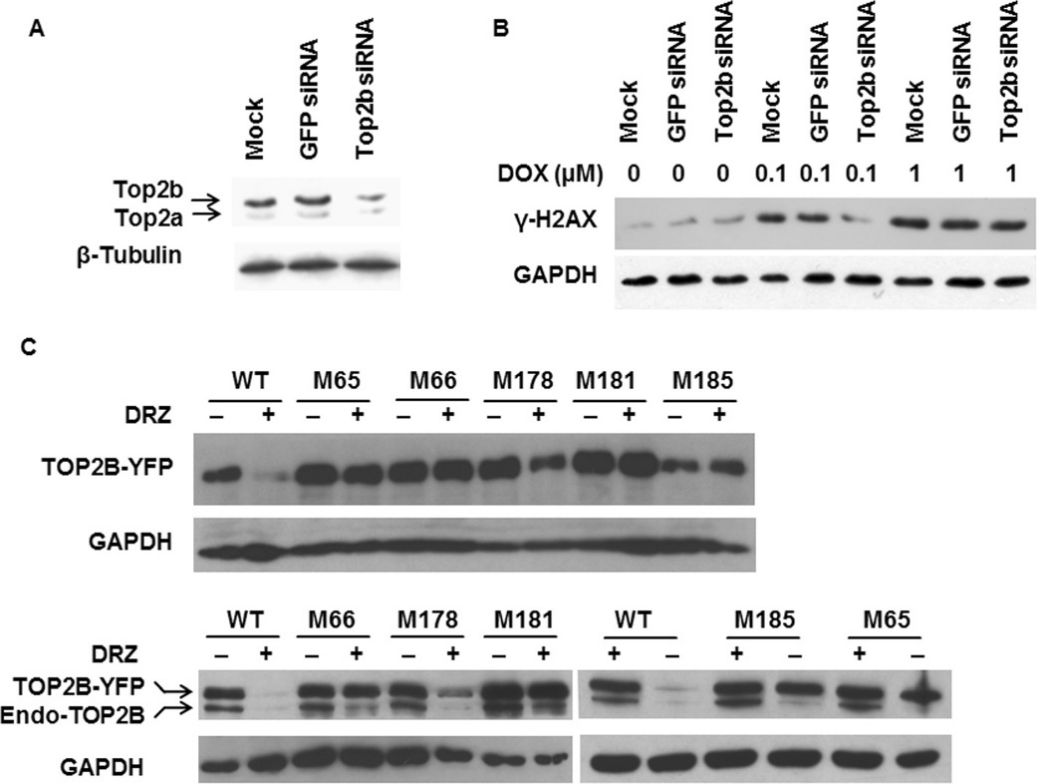
**The effect of N-terminal TOP2B mutants on DRZ-induced TOP2B depletion. (A)** siRNA oligonucleotides against rat Top2b or GFP (control) were transfected into H9C2 cells and Western blot was performed to detect Top2a/b protein level 48 h after transfection. **(B)** Western blot of γ-H2AX in mock-, GFP-siRNA- or TOP2B-siRNA-transfected H9C2 cells incubated with 0.1 or 1 μM DOX for 24 h. siRNA were transfected 24 h before DOX addition. **(C)** HTETOP cells were transiently transfected with plasmids expressing YFP-tagged wild type (WT) or mutated TOP2B carrying single amino acid mutation. DRZ (100 μM) was added 24 h later and Western blot was conducted using antibody specific for YFP (upper panel) or human TOP2B (lower panel) after another 24 h.

To provide a more direct proof of Top2b involvement in the protective effect of DRZ, we manipulated this enzyme’s level. In H9C2 cells, Top2b depletion by means of siRNA (Figure 3A) protected against 0.1 μM DOX-induced DNA damage (Figure 3B) in a similar way as did DRZ (Figure 2D). Top2b knockdown by siRNA was less protective against DSB by 1 μM DOX, consistent with non-enzyme mediated DSB formation by high concentration of DOX [14]. Nevertheless, the reduction of Top2 still may have been an accompanying rather than causative event in the prevention of DOX-induced DSB. To establish a causative link, we took advantage of the previously identified TOP2A variants which confer resistance against DRZ. We focused on five of those variants in the N-terminal domain of Top2A, which is responsible for binding DRZ and other bisdioxopiperazines [26]. Wild-type residues of all five Top2A variants i.e. T49I [27], Y50F [27], R162Q [28], Y165S [29] and L169F [30, 31], are conserved between human TOP2A and TOP2B. The corresponding residues of TOP2B were individually mutated (T65I, Y66F, R178Q, Y181S, and L185F) by site-directed mutagenesis in a vector expressing wild-type human TOP2B with YFP fused at the C-terminus [22]. Plasmids encoding WT or mutated TOP2B were then transiently transfected into human fibrosarcoma-derived HTETOP cells. We employed for this experiment HTETOP cells, as they are easier to transfect and offer the possibility of tetracycline (TET)-mediated depletion of TOP2A [20]. With the exception of R178Q, all mutants were resistant to DRZ-induced depletion when compared to WT TOP2B in Western blot using anti-YFP antibody (Figure 3C, upper panel). This was confirmed by an anti-TOP2B antibody, which also showed the expected depletion of the endogenous WT TOP2B following DRZ exposure (Figure 3C, lower panel).

**Figure 4:**
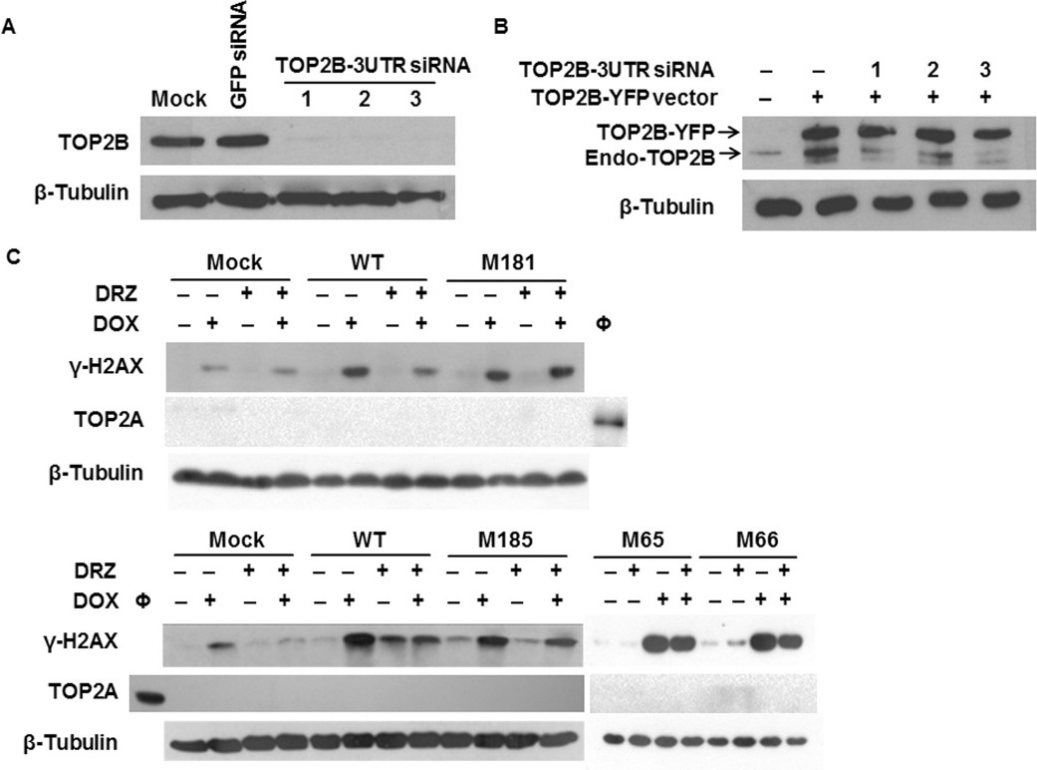
**The effect of N-terminal TOP2B mutants on the DNA-protective effect of DRZ. (A)** and **(B)** HTETOP cells were transfected with siRNA oligonucleotides targeting 3-UTR region of TOP2B mRNA for 48 h. Cells shown in **(B)** were transfected 24 h earlier with plasmids expressing YFP-tagged wild type TOP2B. Both endogenous and YFP-tagged TOP2B protein levels were detected by antibody specific for human TOP2B. **(C)** HTETOP cells were transfected with plasmid expressing WT or mutated YFP-tagged TOP2B. Twenty-four hours later 1 μg/ml TET and 50 nM TOP2B 3-UTR siRNA were added for another 24 h, finally the cells were exposed to 1 μM DOX, 100 μM DRZ alone or in combination (DRZ was given 30 min before DOX) for further 24 h. γ-H2AX accumulation and TOP2A protein level were determined by Western blot**.** Φ: untreated cells, as the positive control for TOP2A detection.

We then investigated if the four TOP2B mutants resistant to DRZ-induced depletion still exhibit protection against DOX-induced DSB. To be able to distinguish between DSB mediated by the transfected TOP2B-YFP and those mediated by endogenous TOP2A or TOP2B, the endogenous TOP2A was removed by pre-treatment with TET and TOP2B was knocked down using siRNA oligos targeting the 3-UTR (Figure 4A). The protein level of TOP2B-YFP expressed from transfected plasmids remained unchanged upon siRNA transfection (Figure 4B) as the 3-UTR of TOP2B is absent from these constructs. DOX-induced DSB formation was much higher in cells depleted of endogenous TOP2A and TOP2B and transfected with wild-type or mutated TOP2B-YFP than in mock-transfected cells. As expected, DRZ attenuated DOX-induced DSB formation in cell transfected with wild-type TOP2B. In contrast, this protection was abolished by mutant M181 (Figure 4C, upper panel) and weakened by mutants M185, M65, and M66 (Figure 4C, lower panel). The above results were consistent with prevention of DOX-induced DSB by DRZ being mediated by the bisdioxopiperazine binding site of TOP2B.

### DRZ may deplete TOP2A in addition to TOP2B

In contrast to post-mitotic cells such as cardiomyocytes, proliferating cells such as tumor cells express TOP2A or both isoforms rather than TOP2B alone. As any DRZ interference with TOP2A could affect the TOP2A-mediated therapeutic response to anthracyclines [19], we investigated the effect of DRZ on TOP2A and on DOX-induced DSB mediated by this isoform.

**Figure 5:**
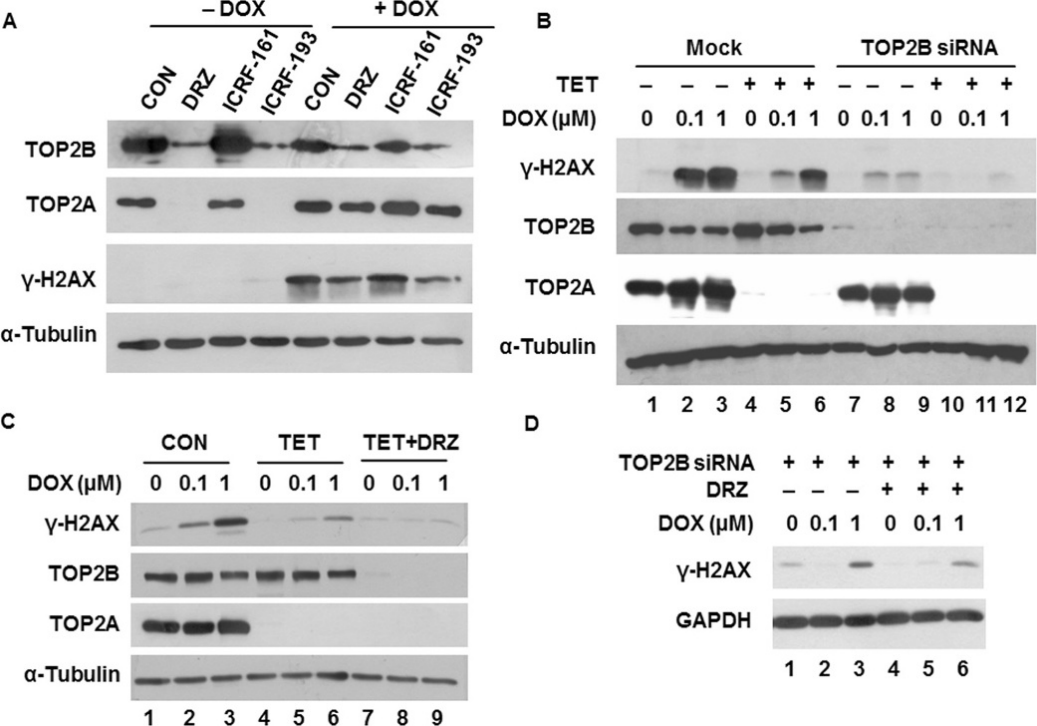
**The individual contributions of TOP2A and TOP2B to DOX-induced DNA damage and to its prevention by DRZ. (A)** HTETOP cells were treated for 24 h with DRZ, its analogue ICRF-161 or ICRF-193, alone or combined with 1 μM DOX. TOP2B, TOP2A and γ-H2AX protein levels were determined by Western blot. **(B)** Western blot of γ-H2AX, TOP2B and TOP2A in mock- or TOP2B siRNA-transfected HTETOP cells incubated with 0.1 or 1 μM DOX for 24 h. TOP2A was depleted from HTETOP cells by 24 h pre-incubation with 1 μg/ml TET, followed by TOP2B siRNA transfection. DOX was added 24 h later following transfection. **(C)** and **(D)** HTETOP cells were pre-treated with 1 μg/ml TET **(C)** or TOP2B siRNA **(D)** for 24 h, followed by 24 h exposure to DOX alone or in combination with 100 μM DRZ. DRZ was administered 30 min before DOX. γ-H2AX, TOP2A and TOP2B protein levels were determined by Western blot.

We previously reported DRZ-induced TOP2A depletion in HTETOP cells, which was proteasome-independent and accompanied by TOP2A mRNA reduction [14]. The depletion most likely required direct TOP2A-DRZ interaction, as it was absent from cells treated with the non-binding DRZ analogue ICRF-161 (Figure 5A). Likewise, ICRF-161 did not reduce DOX-induced DSB accumulation, in contrast to DRZ and ICRF-193 (Figure 5A).

Does DRZ affect DOX-induced DSB formation mediated by TOP2A? To answer this question we first assessed the contributions of the individual TOP2 isoforms to DOX-induced DNA damage. DSB were barely detectable in DOX-treated HTETOP cells depleted of TOP2B by means of siRNA and of TOP2A by TET (lanes 11–12 vs. 2–3 in Figure 5B). The DSB level was higher in DOX-treated cells expressing TOP2A but not TOP2B (lanes 11–12 vs. 8–9 Figure 5B). Likewise, DOX-induced DSB were reduced by TET-mediated TOP2A removal in cells expressing TOP2B (lanes 5–6 vs. 2–3 in Figure 5B). These results demonstrated that both TOP2 isoforms mediated DOX-induced DSB.

We then assessed the effect of DRZ on the DOX-induced DSB formation mediated by each TOP2 isoform. The formation of DOX-induced DSB in HTETOP cells depleted of TOP2A by TET was reduced by DRZ (lanes 8–9 vs. 5–6 in Figure 5C). This was accompanied by TOP2B depletion and it was consistent with the involvement of TOP2B in DOX-induced DNA damage. The effect of DRZ on DOX-induced and TOP2A-mediated DSB was assessed in HTETOP cells depleted of TOB2B by means of siRNA (Figure 5D). The level of DOX-induced DSB in these cells was further reduced by DRZ (lanes 6 vs. 3 in Figure 5D), indicating the contribution of TOP2A to the protective effect.

**Figure 6:**
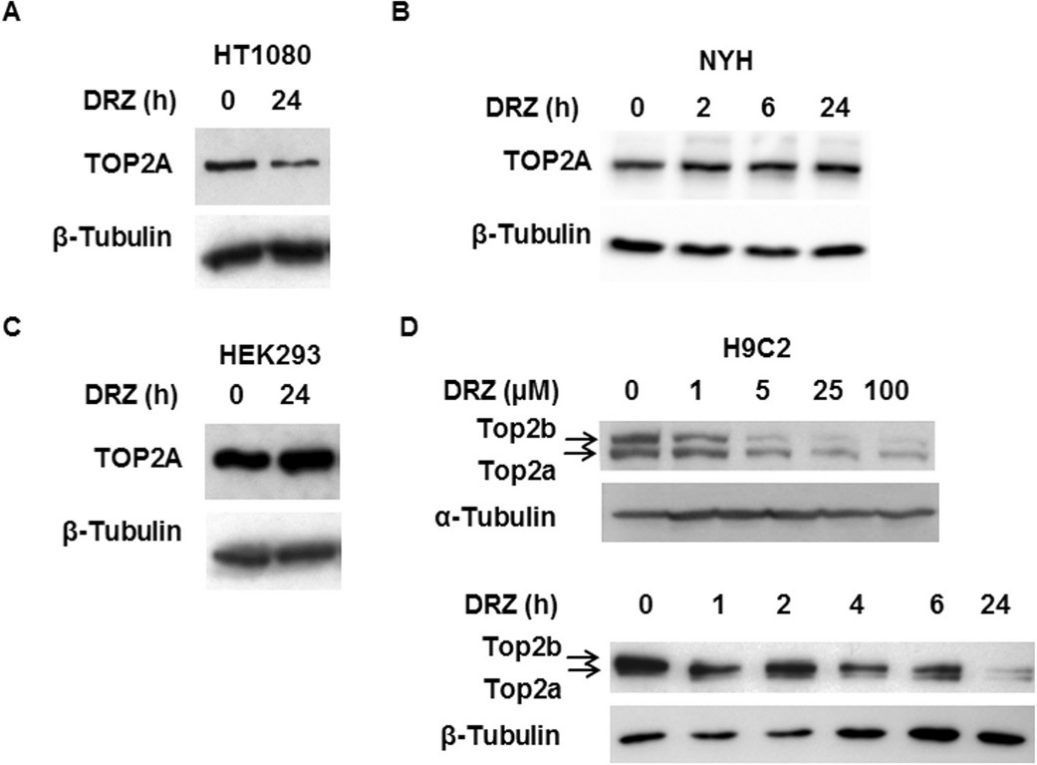
**TOP2A protein levels in response to DRZ.** 24 h incubation with 100 μM DRZ in HT1080 **(A)**, NYH **(B)**, HEK293 **(C)**, and in differentiated H9C2 cells. The lower panel of **(D)** additionally shows the effect of incubation time in H9C2 cells.

How general a phenomenon is the DRZ-triggered TOP2A depletion? DRZ (100 μM, 24 h) reduced the level of TOP2A protein in fibrosarcoma-derived HT1080 (Figure 6A), but neither in lung cancer-derived NYH cells (Figure 6B), nor in human embryonic cells HEK293 (Figure 6C). In contrast, DRZ depleted both Top2a and Top2b in a time – and concentration-dependent manner from differentiated H9C2 cells, which re-express TOP2A following the addition of fresh serum (Figure 6D).

## Discussion

Our study contributes to the mechanistic understanding of the prevention of anthracycline-induced heart failure by DRZ. Simultaneously, it suggests mechanistic explanation for the inconsistent and sometimes contradictory reports of DRZ impact on anthracycline anticancer therapies. In the light of our data, these effects may be mediated by DRZ-triggered depletion of TOP2B.

The long-held view that cardioprotection involves iron-chelation by the EDTA-like product of the enzymatic DRZ hydrolysis ADR-925 has been recently questioned. Indeed, ADR-925 does not protect cardiomyocytes against anthracyclines [7], while prevention of its generation from DRZ does not abolish the cardioprotective effect [8]. These observations implicate DRZ itself as the cardioprotective compound. As several previous studies demonstrated TOP2B depletion by DRZ and its analogues [14–16], we first verified these observations and investigated the underlying mechanism in more detail. We found DRZ capable of TOP2B depletion in all cell lines tested, including differentiated cardiomyocytes. In agreement with cell line data, we detected transient depletion of Top2b by DRZ in the mouse heart tissue, which constitutes a first report of this phenomenon *in vivo*.

We provide several lines of complementary evidence for DRZ-mediated TOP2B depletion being protective against anthracycline-induced DNA damage. The reduction of DOX-induced DNA damage by DRZ was associated with TOP2B depletion and it was similar to that observed upon TOP2B removal by siRNA. That this reduction was indeed mediated by the TOP2B removal was evidenced first of all by the differential effect of DRZ analogues. Thus, the reduction of DOX-induced DNA damage was conferred by TOP2B-binding and -depleting (DRZ, ICRF-193), but not by the non-binding and non-depleting bisdioxopiperazine ICRF-161. In contrast to DRZ, ICRF-161 is not protective against DOX-induced heart damage [25]. Taken together with our experiments, a bisdioxopiperazine’s cardioprotective effect seems therefore to be determined by its capability to deplete TOP2B rather than to chelate iron. Indirectly, these results support the emerging evidence that anthracycline-induced cardiotoxicity is driven by TOP2B-mediated DNA damage rather than by oxidative stress [17].

To verify these conclusions mechanistically for the clinically relevant bisdioxopiperazine DRZ, we took advantage of the existing TOP2 DRZ resistance mutants. Strikingly, although not surprisingly, these mutants localize to the bisdioxopiperazine binding site in the N-terminal domain of TOP2 [26]. All but one mutant prevented the depletion of TOP2B. Simultaneously, these mutants reduced the protective effect of DRZ against DOX-induced DNA damage. This result constitutes currently the strongest evidence that DRZ prevents DNA damage by depleting TOP2B, which is mediated through the bisdioxopiperazine binding site.

Taken together with the protective effect of a genetic Top2b deletion against DOX-induced cardiotoxicity [17], our results suggest the following model of DRZ-mediated cardioprotection. DRZ depletes cardiac TOP2B, thereby reducing the substrate for DOX poisoning. Subsequent administration of an anthracycline results in lower levels of DNA damage than without DRZ pretreatment, i.e. in the presence of normal TOP2B levels. The depletion of TOP2B is transient, which reduces the risk or at least the duration of any adverse effects of this enzyme’s deficiency. Although we have failed to measure DSB in vivo, this model is fully consistent with the reducing effect of DRZ on Top2b levels and Top2b-mediated DSB in cultured rat cardiomyocytes. Moreover, it is consistent with the observation that DRZ is no longer cardioprotective when given after anthracyclines [32], i.e. when DNA damage has already occurred.

The depletion of TOP2B by DRZ characterized in this study may also help to resolve the persisting concern of this drug’s interference with antitumor efficacy of anthracyclines. This concern has its seeds in a randomized, double-blind study of patients with advanced breast cancer treated with fluorouracil, DOX, and cyclophosphamide with or without DRZ [3]. Although time to progression and survival were not significantly different, DRZ-treated patients showed a lower tumor response rate, in addition to the expected reduction of cardiotoxicity. The reduction of the response rate is no longer statistically significant in the latest update of the pertinent Cochrane meta-analysis, but a trend remains [33].

Based on the growing evidence of TOP2B depletion by DRZ and its molecular characterization in the present study, we would like to argue that DRZ may lower the response rate by depleting TOP2B. Although the anti-tumor effects of TOP2 poisons are usually attributed to TOP2A rather than TOP2B, the application of TOP2A level as a therapeutic predictor has been unsuccessful [34]. Quite the contrary, breast cancer survival can be predicted by TOP2B rather than TOP2A expression level [35]. This could be explained by the TOP2B expression in >90% of cells in breast tumors. In contrast, TOP2A expression is restricted to the much smaller subset of proliferating (i.e. Ki67-positive) cells of breast tumors [35]. A predominance of TOP2B in the combined TOP2A/TOP2B activity has been also described in ovarian tumors [36].

As confirmed in the present study, both TOP2 isoforms mediate DOX-induced DNA damage in tumor cells. DRZ-driven depletion of TOP2B would be expected to reduce this damage and thereby the elimination of tumor cells. The DRZ-driven depletion of TOP2B is apparently transient, which would likely minimize any long-term effects deleterious to tumor cell survival. In consequence, DRZ would be expected to reduce tumor shrinkage, a key component of the tumor response. These considerations are in agreement with the reduction of therapy response by DRZ detected in breast cancer patients [3].

Importantly, the reduction of tumor response by DRZ has been observed when DRZ was applied beginning with the first therapy course [3]. When given to breast cancer patients first when they had reached a cumulative DOX dose of 300 mg/m2, DRZ almost doubled the survival [6]. This effect is more difficult to explain in mechanistic terms, but it is important to remember that DRZ has some weak antitumor effects [14]. These effects could become manifest only when a certain tumor shrinkage has been achieved. In other words, initially better tumor shrinkage in the absence of DRZ could sensitize the tumor to this drug’s anti-tumor effect when included into later therapy courses. Incidentally, this situation corresponds to the currently approved application of DRZ in breast cancer patients.

Finally, we verify and expand the original observation that DRZ may deplete TOP2A in addition to TOP2B [14]. This effect may be mediated by a mechanism different from that of TOP2B depletion, as it is independent from the proteasome activity but accompanied by lower TOP2A mRNA levels [14]. The depletion of TOP2A is less ubiquitous in comparison to TOP2B, as it has been detected only in some cell lines. The discrepancy of DRZ effects on TOP2A in H9C2 cells in this and a previous work [15] may have been caused by our longer incubation times and by utilization of differentiated cells which express lower TOP2A levels. It is tempting to speculate that TOP2A depletion could account for the only side-effects of DRZ detected in current meta-analyses, i.e. low white blood count at nadir and anemia [33]. Indeed, TOP2A is expressed predominantly in proliferating compartments such as bone marrow [18, 37]. The effects of DRZ on the stability and expression of TOP2 isoforms in normal tissues deserve detailed investigations.

## Conclusions

DRZ may prevent doxorubicin-induced DNA damage through depleting TOP2A and TOP2B. It prevents anthracycline-induced heart failure via TOP2B degradation in heart tissues rather than via iron chelation. The depletion of TOP2B and TOP2A in tumors may suggest an explanation for the reported DRZ interference with cancer response to anthracyclines.

## Abbreviations

DRZ: Dexrazoxane
TOP2A: Topoisomerases IIα
TOP2B: Topoisomerases IIβ
DSB: Double strand breaks
HF: Heart failure
DOX: Doxorubicin
YFP: Yellow fluorescent protein
GFP: Green fluorescent protein
FPKM: Fragment Per Kilobase of exon per Million fragments mapped
WT: Wild type.
